# Molecular determinants of resurgent sodium currents mediated by Navβ4 peptide and A-type FHFs

**DOI:** 10.3389/fnmol.2024.1433981

**Published:** 2024-10-02

**Authors:** Yucheng Xiao, Yanling Pan, Jingyu Xiao, Theodore R. Cummins

**Affiliations:** ^1^Biology Department, School of Science, Indiana University Indianapolis, Indianapolis, IN, United States; ^2^School of Engineering, Purdue University, West Lafayette, IN, United States

**Keywords:** sodium channels, resurgent currents, Navb4, fibroblast growth factor homologous factor, electrophysiology, mutagenesis

## Abstract

**Introduction:**

Resurgent current (*I_NaR_*) generated by voltage-gated sodium channels (VGSCs) plays an essential role in maintaining high-frequency firing of many neurons and contributes to disease pathophysiology such as epilepsy and painful disorders. Targeting *I_NaR_* may present a highly promising strategy in the treatment of these diseases. Navβ4 and A-type fibroblast growth factor homologous factors (FHFs) have been identified as two classes of important *I_NaR_* mediators; however, their receptor sites in VGSCs remain unknown, which hinders the development of novel agents to effectively target *I_NaR_*.

**Methods:**

Navβ4 and FHF4A can mediate *I_NaR_* generation through the amino acid segment located in their C-terminus and N-terminus, respectively. We mainly employed site-directed mutagenesis, chimera construction and whole-cell patch-clamp recording to explore the receptor sites of Navβ4 peptide and FHF4A in Nav1.7 and Nav1.8.

**Results:**

We show that the receptor of Navβ4-peptide involves four residues, N395, N945, F1737 and Y1744, in Nav1.7 DI-S6, DII-S6, and DIV-S6. We show that A-type FHFs generating *I_NaR_* depends on the segment located at the very beginning, not at the distal end, of the FHF4 N-terminus domain. We show that the receptor site of A-type FHFs also resides in VGSC inner pore region. We further show that an asparagine at DIIS6, N891 in Nav1.8, is a major determinant of *I_NaR_* generated by A-type FHFs in VGSCs.

**Discussion:**

Cryo-EM structures reveal that the side chains of the critical residues project into the VGSC channel pore. Our findings provide additional evidence that Navβ4 peptide and A-type FHFs function as open-channel pore blockers and highlight channel inner pore region as a hotspot for development of novel agents targeting *I_NaR_*.

## Introduction

Voltage-gated sodium channels (VGSCs) can generate classic sodium current and atypical sodium current named resurgent current (*I_NaR_*) during action potentials. While the former is evoked by membrane depolarization, *I_NaR_* activates during the action potential repolarization following a brief depolarization. *I_NaR_* was first described in isolated Purkinje neurons (Raman and Bean, 1997) and since has been identified in more than 20 types of neurons in the central and peripheral nervous systems such as cerebellum, brainstem, trigeminal ganglia, and dorsal root ganglion (DRG) neurons (Afshari et al., 2004; Cummins et al., 2005; Enomoto et al., 2006; Kim et al., 2010; Lewis and Raman, 2014). Compared to classic sodium current, *I_NaR_* is generally small, but it is essential in many neurons to maintain high-frequency firing, spontaneous firing, and action potential duration (Raman and Bean, 1997; Raman et al., 1997; Jarecki et al., 2010; Xiao et al., 2022).

*I_NaR_* generation requires mediation of other auxiliary subunits that compose VGSC complexes. To date, at least two subunits Navβ4 and A-type fibroblast growth factor homologous factors (FHFs) have been reported to mediate *I_NaR_* generation (Grieco et al., 2005; White et al., 2019; Xiao et al., 2022). Navβ4 interacts with VGSC α subunit via an N-terminal cysteine in its extracellular Ig domain (Gilchrist et al., 2013), and A-type FHFs compose VGSC complexes via their β-trefoil core domain docking at VGSC α subunit C-terminus (Goldfarb, 2005). However, *I_NaR_* generation does not result from these domains but seemingly from the amino acid segments located within Navβ4 C-terminus and A-type FHF N-terminus, respectively (Aman and Raman, 2023). Studies have shown that *I_NaR_* can be fully reconstituted by the synthetic peptide containing the sequence Navβ4 K154 – K167 or FHF4A A2 – K21 in heterologous systems (Grieco et al., 2005; Xiao et al., 2022). These two peptides exhibit low amino acid sequence identity to each other, but they are both proposed to mediate *I_NaR_* through a relief-of-open-channel-block mechanism. Mechanically, the peptides are proposed to act as an intracellular open-channel blocker and functionally compete with the classic VGSC inactivation particle during depolarization and channel opening. Upon repolarization, the blocker unbinds, and the channel reconducts, producing *I_NaR_* (Cannon and Bean, 2010).

*I_NaR_* has been observed to be dysregulated in both acquired and inherited disorders of neuronal excitability. They are enhanced by painful animal toxins, inflammatory mediators, and many mutations in VGSCs associated with epilepsy, pain, myotonia and cardiac arrhythmias (Grieco and Raman, 2004; Jarecki et al., 2010; Tan et al., 2014; Patel et al., 2016; Xiao et al., 2019). Computer simulations and pharmacological studies further indicate that increased *I_NaR_* may be the major contributor to neuronal hyperexcitability (Khaliq et al., 2003; Jarecki et al., 2010; Raman, 2023). Intriguingly, the hyperexcitability can be reversed by selective inhibition of *I_NaR_* or by knockdown of Navβ4 and FHF4A (Bosch et al., 2015; Patel et al., 2016; Xiao et al., 2019). These findings strongly suggest that targeting *I_NaR_* may present a highly promising strategy in treatment of these diseases. However, the receptor sites of Navβ4 and A-type FHFs in VGSCs that determine *I_NaR_* remain unknown, which has hindered the development of novel agents to effectively target *I_NaR_*.

In this study we focused on Nav1.7 and Nav1.8, two VGSC subtypes playing essential roles in pain sensation and believed to be ideal targets for development of novel agents to treat pain (Cox et al., 2006; Cummins et al., 2007; Dib-Hajj et al., 2010). We found that the two putative mediators, Navβ4 peptide and FHF4A peptide, can differentially influence gating properties of VGSCs. We found that three residues in Nav1.7 pore region (N395 in DI-S6, and F1737 and Y1744 in DIV-S6) are crucial for Navβ4 peptide to mediate *I_NaR_*. We also found that mutating N891 and F1710 in Nav1.8, corresponding to S969 and F1737 in Nav1.7, respectively, renders Nav1.8 resistant to FHF4A. Cryo-electron microscopy (cryo-EM) structures of Nav1.7 and Nav1.8 reveal that the side chains of several of critical residues project into VGSC channel pore. Therefore, our findings not only provide evidence that both Navβ4 peptide and FHF4A peptide function as open-channel pore blockers, but also highlight channel pore region as a hotspot to develop novel agents to effectively target *I_NaR_*.

## Materials and methods

### Plasmids, sodium channel constructs, and mutagenesis

Human FHF2B sequence was subcloned into pmTurquoise2-N1 vector as described by Barbosa et al. (2017). The pCMV6-AC-GFP plasmid encoding human FHF4A was purchased from Origene USA Technologies, Inc. (Rockville, MD). The cDNA construct encoding human Nav1.7 and human Nav1.8 were subcloned into a pcDNA3.1 expression vector, respectively. All mutations in Nav1.7 and Nav1.8 were constructed using the QuikChange XL (Stratagene) mutagenesis kit following the manufacturer’s instructions. The cDNAs encoding three FHF4A/FHF2A chimeric proteins were synthesized from GenScript USA, Inc. (Piscataway, NJ) and subcloned into the pCMV6-AC-GFP vector. All mutations and chimeric cDNAs were confirmed by sequencing.

### Peptides

Three peptides (Navβ4 peptide, KKLITFILKKTREK; FHF4A peptide, AAAIASGLIRQKRQAREQHW; scrambled peptide, KAREAQRASRAIEQLRSAKI) were synthesized from Biopeptide Co., Inc. (CA, United States).

### Cell culture and transfection

HEK293 cells and ND7/23 cells were grown under standard tissue culture conditions (5% CO_2_ and 37°C) in DMEM supplemented with 10% FBS, 1% penicillin and 1% streptomycin. Using Invitrogen Lipofectamine 2000, human Nav1.7 R1599P (rpNav1.7) and the mutant constructs were transiently co-transfected with Navβ1 and Navβ2 into HEK293 cells. The constructs human Nav1.8 and mutants were transiently co-transfected with FHF2B or FHF4A into ND7/23 cells. The lipofectamine-DNA mixture was added to the cell culture medium and left for 3 h after which the cells were washed with fresh medium. Cells with green fluorescent protein fluorescence were selected for whole-cell patch-clamp recordings 36–72 h after transfection. ND7/23 cells do not express endogenous Nav1.8 currents but do express endogenous TTX-sensitive sodium currents (John et al., 2004; Lee et al., 2019). Therefore, transfected ND7/23 cells were pretreated with 500 nM TTX to isolate the recombinant Nav1.8 currents.

### Electrophysiological recordings

Whole-cell voltage-clamp recordings were performed at room temperature (~21°C) using an EPC-10 amplifier and the Pulse program (HEKA Electronics). Fire-polished electrodes (0.7–1.0 MΩ) were fabricated from 1.7 mm capillary glass using a P-97 puller (Sutter Instruments), and the tips were coated with sticky wax (KerrLab) to reduce electrode capacitance and enable increased series resistance compensation. The pipette solution contained (in mM) 140 CsF, 1.1 EGTA, 10 NaCl, and 10 HEPES, pH 7.3. The bathing solution was (in mM) 130 mM NaCl, 30 mM TEA chloride, 1 mM MgCl_2_, 3 mM KCl, 1 mM CaCl_2_, 10 mM HEPES, and 10 mM d-glucose, pH 7.3 (adjusted with NaOH). The liquid junction potential for these solutions was <8 mV; data were not corrected to account for this offset. The offset potential was zeroed before contacting the cell. After establishing the whole-cell recording configuration, the resting potential was held at −120 or −100 mV for 5 min to allow adequate equilibration between the micropipette solution and the cell interior. Linear leak subtraction, based on resistance estimates from four to five hyperpolarizing pulses applied before the depolarizing test potential, was used for all voltage clamp recordings. Membrane currents were usually filtered at 5 kHz and sampled at 20 kHz. The average series resistance was 2.4 ± 0.8 MΩ (*n* = 216) during whole cell recordings prior to compensation. Voltage errors were minimized using 80–95% series resistance compensation, and the capacitance artifact was canceled using the computer-controlled circuitry of the patch clamp amplifier. Navβ4 peptide (10 μM – 1 mM) and FHF4A peptide (10 μM – 1 mM) were applied to intracellular solution. The concentration-dependent curves were fitted to a hill equation: Y = Bottom + (Top-Bottom)/(1 + 10^((LogEC50-X)*HillSlope)).

#### Steady-state activation

Families of sodium currents were induced by 50-ms depolarizing steps to various potentials ranging from −120 (or −100) to +40 mV in 5-mV increments. The conductance was calculated using the equation *G*(Nav) = *I*/(*V* - *Vrev*) in which *I*, *V*, and *Vrev* represent inward current value, membrane potential, and reversal potential, respectively.

#### Steady-state inactivation

Voltage dependence of steady-state inactivation was estimated using a standard double pulse protocol in which sodium currents were induced by a 50-ms depolarizing potential of 0 mV following a 500-ms prepulse at potentials that ranged from −150 to +10 mV with a 10-mV increment. Currents were plotted as a fraction of the maximum peak current. To obtain the midpoint voltages (*V_1/2_*) and slope factor (*k*), the curves of both steady-state activation and inactivation were fitted to a Boltzmann function.

*I_NaR_*: *I*_NaR_ was elicited by repolarizing voltage steps from +20 to −80 mV for 100 ms (or 200 ms) in −5 mV increments, following a 20-ms depolarizing potential of +30 mV. To avoid contamination from tail currents, Navβ4 peptide-induced Nav1.7 *I*_NaR_ was measured after 3.0 ms into the repolarization pulse, FHF4A or FHF4A peptide-induced Nav1.8 *I*_NaR_ was measured after 20 ms into the repolarization pulse. The relative *I*_NaR_ in Nav1.7 and Nav1.8 was calculated by normalizing to the peak transient current elicited at −10 or 0 mV.

### Fluorescence imaging

The constructs FHF4A and FHF4A/FHF2A chimeras (3 μg) were transiently transfected into ND7/23 cells using Invitrogen Lipofectamine 2000 as described above. Fluorescence imaging was performed using ThorCam^™^ software (Thorlabs, Inc.) 36 h after transfection. Images were analyzed with ImageJ software (NIH) and corrected mean cell fluorescence was calculated in Excel (Microsoft) by applying measurements obtained from image analysis using the equation: CMCF = mean intensity – mean fluorescence of background recordings. The intensity of fluorescence of the whole ND7/23 cell was measured.

### Experimental design and statistical analysis

The acquisition of control and experimental data was randomized. Data were analyzed using the software programs PulseFit (HEKA) and GraphPad Prism 5.0 (GraphPad Software, Inc., San Diego, CA). All data are shown as mean ± S.E. The numbers of separate experimental cells and experimental groups are presented as *n* and *N*, respectively. Statistical analysis was performed by two-tailed Student’s t test or one-way ANOVA test with post doc Tukey test. The Student’s *t* test was used if not specified in the sections of Results and Discussion. *p* < 0.05 indicated a significant difference.

## Results

### The R1599P mutation enhanced Nav1.7 to produce Navβ4 peptide *I_NaR_*

**Figure 1:**
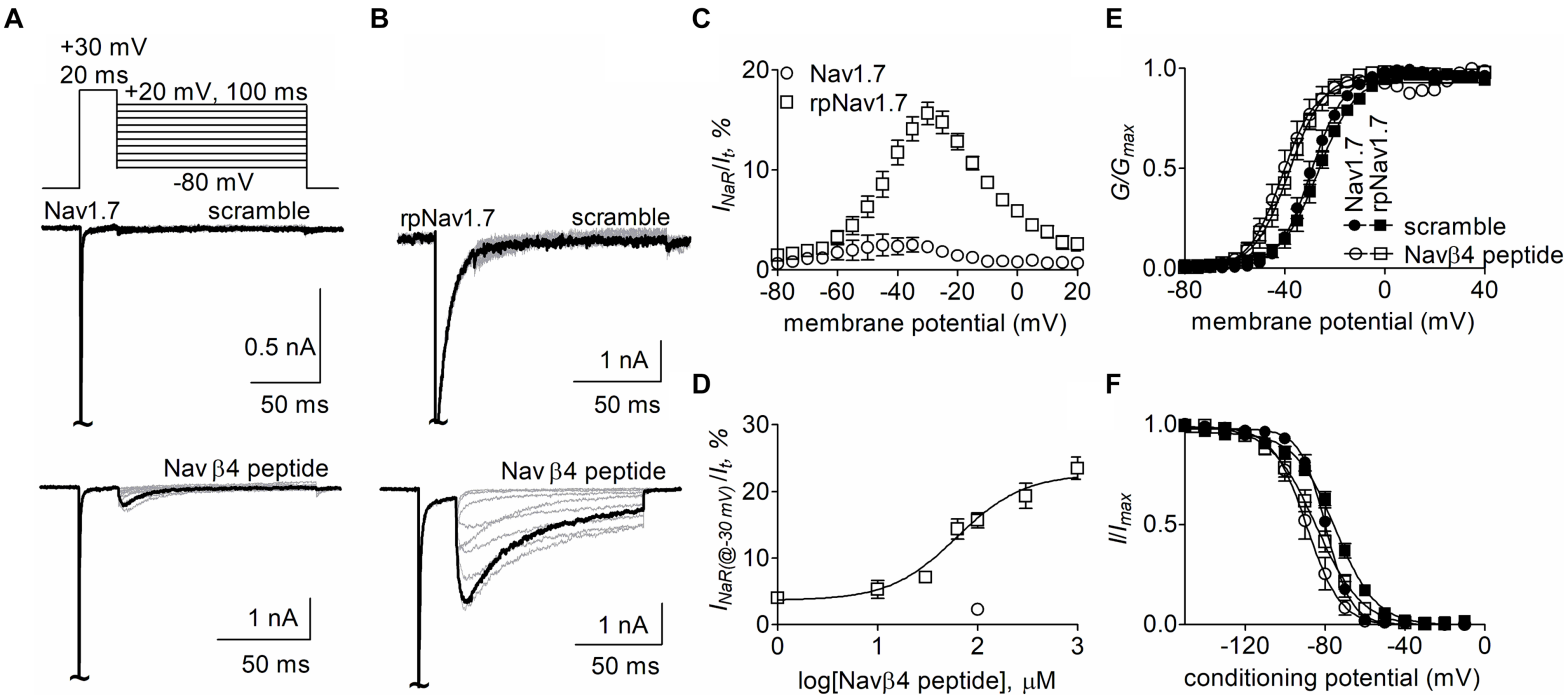
Navβ4 peptide mediated *I_NaR_* generated by Nav1.7 or rpNav1.7 co-expressed with Navβ1 and Navβ2 in HEK293 cells. **(A,B)** Family of representative current traces were elicited from Nav1.7 **(A)** or rpNav1.7 **(B)** in the presence of 100 μM scrambled peptide (top) or Navβ4 peptide (bottom) by a standard *I_NaR_* protocol (*inset*), in which cells were applied by 100-ms repolarizing voltage steps from +20 mV to −80 in −10 mV increments, following a 20-ms depolarizing potential of +30 mV. The current trace highlighted was elicited at −30 mV. **(C)** Voltage dependence of the relative *I_NaR_* mediated by 100 μM Navβ4 peptide (*n* = 14). *I_NaR_* was normalized to the peak transient current elicited at −10 mV. **(D)** Concentration-response curve of Navβ4 peptide. Each data point comes from 3 to 14 separate cells. **(E)** Effect of 100 μM Navβ4 peptide on steady-state activation. Families of sodium currents were induced by 50-ms depolarizing steps to various potentials ranging from −80 to +40 mV in 5-mV increments. **(F)** Effect of 100 μM Navβ4 peptide on steady-state inactivation. Steady-state inactivation was estimated using a standard double pulse protocol in which sodium currents were induced by a 50-ms depolarizing potential of −10 mV following a 500-ms prepulse at potentials that ranged from −150 to +10 mV with a 10-mV increment. Cells were held at −120 mV. For Nav1.7, V_1/2(activation)_: scramble, −29.3 ± 1.6 mV (*n* = 6) *vs* Navβ4 peptide, −40.5 ± 2.8 (*n* = 8), *p* < 0.01; V_1/2(inactivation)_: scramble, −79.7 ± 1.7 mV (*n* = 6) *vs* Navβ4 peptide, −88.8 ± 3.0 (*n* = 8), *p* < 0.05. For rpNav1.7, V_1/2(activation)_: scramble, −27.0 ± 1.9 mV (*n* = 6) *vs* Navβ4 peptide, −38.7 ± 2.2 (*n* = 9), *p* < 0.01; V_1/2(inactivation)_: scramble, −75.0 ± 1.5 mV (*n* = 5) *vs* Navβ4 peptide, −84.5 ± 2.8 (*n* = 9), *p* < 0.05. “~“indicates where the transient peak current was truncated to display *I_NaR_* more clearly.

The goal of this study is to determine the critical residues in VGSCs generating Navβ4 peptide and FHF4A peptide mediated *I_NaR_*. Our previous work has shown that wild type (WT) Nav1.7 exhibits a limited capability to generate Navβ4 peptide *I_NaR_*, although paroxysmal extreme pain disorder (PEPD) mutations that impair inactivation can substantially enhance Nav1.7 *I_NaR_* (Theile et al., 2011). To increase the capability, here we replaced the outermost gating charge residue R1559 with proline in Nav1.7 DIV-S4 because the corresponding mutation R1448P in Nav1.4 is reported to substantially enhance *I_NaR_* generation (Jarecki et al., 2010) and the R1448P mutation is less likely to directly alter the interaction of Navβ4 peptide with the Nav1.7 pore region than many of the PEPD mutations. The Nav1.7 R1599P construct is referred to as rpNav1.7 hereinafter. The rpNav1.7 construct was transiently co-transfected with Navβ1 and Navβ2 into HEK293 cells. The synthetic Navβ4 peptide (KKLITFILKKTREK) at desired concentrations was applied in the intracellular solution. In Figures 1A,B, Navβ4 peptide (100 μM) enabled rpNav1.7 to produce large *I_NaR_*, but no *I_NaR_* was elicited in the presence of 100 μM scrambled peptide (KAREAQRASRAIEQLRSAKI). The *I_NaR_* displayed a rapid onset and rapid decay kinetics, peaked at −30 mV, and could be observed at step repolarization voltages ranging from −55 to +10 mV. The average *I_NaR_* amplitude elicited at −30 mV was 15.7% ± 1.1% of the peak transient current (Figures 1C,D), which is fivefold larger than that observed with WT Nav1.7 (2.3% ± 0.5%, *p* < 0.0001). Navβ4 peptide also caused a hyperpolarizing shift of steady-state activation and inactivation (Figures 1E,F; Table 1). Navβ4 peptide mediated *I_NaR_* fluxing through rpNav1.7 in a concentration-dependent manner. Fitting the Hill equation to the data yielded an EC_50_ value of 61.8 ± 1.3 μM (Figure 1D). According to the concentration-dependent curve, the effect of Navβ4 peptide on *I_NaR_* generation did not saturate at 100 μM. Therefore, we chose this concentration to measure the impact of residues in VGSCs on Navβ4 peptide *I_NaR_*.

**Table 1:** Gating properties of rpNav1.7 and the mutants in the presence of 100 μM Navβ4 peptide.

	rpNav1.7	Y392K	N395K	L398K	V947K	F1737K	Y1744K
Activation (V_1/2_, mV)	−38.7 ± 2.2 (9)	−32.3 ± 4.1 (5)	−39.1 ± 2.0 (5)	−34.8 ± 4.7 (6)	−37.4 ± 1.3 (8)	−16.1 ± 2.1^***^ (7)	−18.9 ± 3.2^***^ (9)
Inactivation (V_1/2_, mV)	−84.5 ± 2.8 (9)	−85.6 ± 1.6 (4)	−78.7 ± 2.4 (5)	−84.5 ± 4.6 (6)	−77.8 ± 1.5 (9)	−77.7 ± 2.4 (7)	−89.2 ± 2.0 (9)
*I_NaP_*, %	7.1 ± 3.0 (9)	7.4 ± 1.6 (5)	16.1 ± 4.9^*^ (5)	11.4 ± 5.0 (6)	9.5 ± 3.4 (8)	2.4 ± 1.3^***^ (8)	4.3 ± 1.6^**^ (9)

Midpoint voltages of the steady-state activation and inactivation curves in Figure 3 were determined with a standard Boltzmann distribution fit. Persistent current (*I_NaP_*) measured at the end of the 50-ms pulse of −10 mV was normalized to the peak transient current. The number of separate cells tested is indicated in parentheses. *p < 0.05; **p < 0.01; ***p < 0.0001 *vs* Nav1.7.

### Effects of residues in Nav1.7 pore region on Navβ4 peptide *I_NaR_*

**Figure 2:**
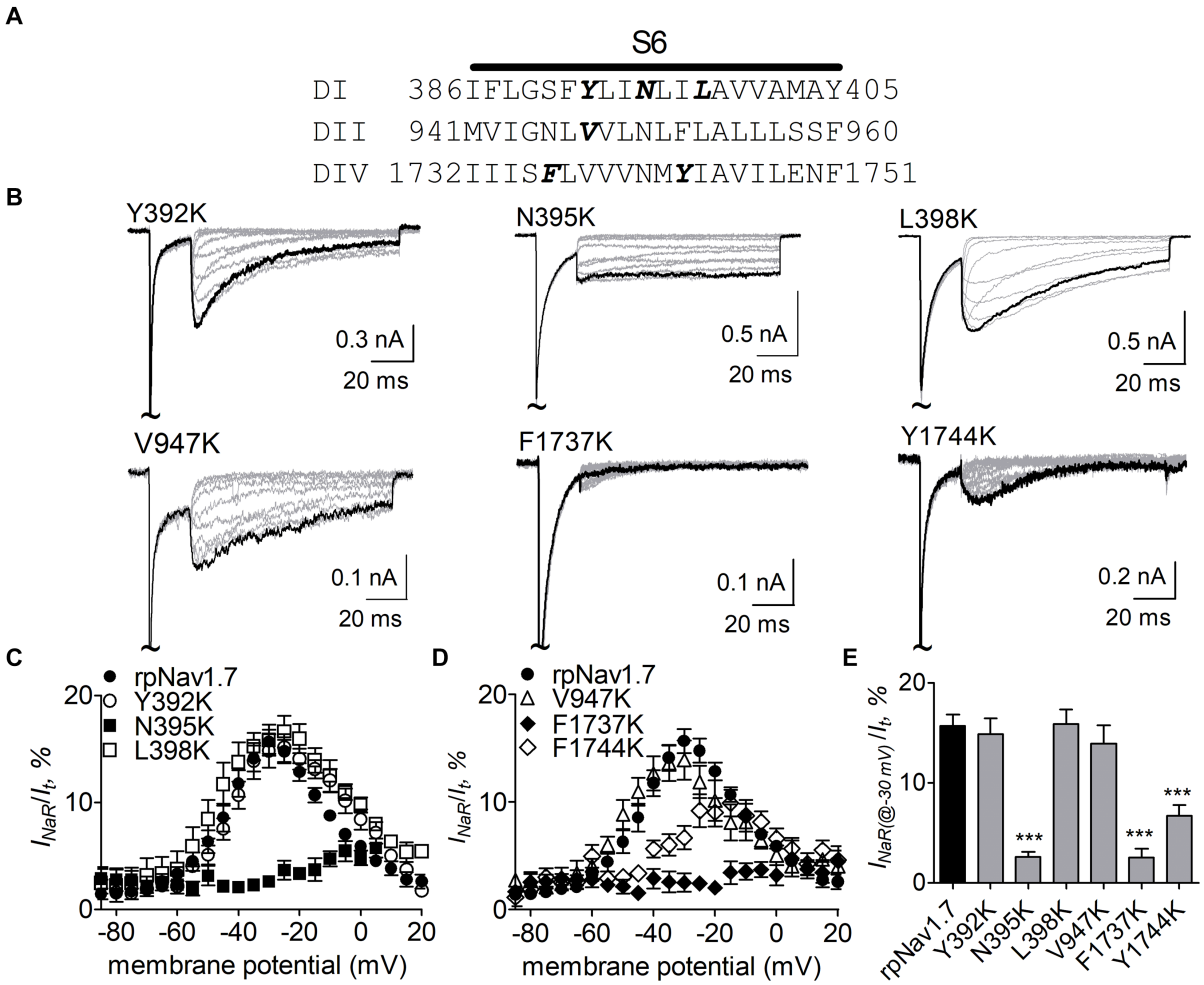
Effects of mutations in rpNav1.7 on Navβ4 peptide *I_NaR_*. **(A)** Amino acid sequence of the S6 segments of Nav1.7-DI, DII and DIV. The residues of interest are highlighted in bold. **(B)** Typical *I_NaR_* traces generated by the rpNav1.7 mutants Y392K, N395K, L398K, V947K, F1737K, and Y1744K. *I_NaR_* were elicited by a protocol shown in Figure 1A (*inset*). The current trace elicited at −30 mV was highlighted. The concentration of Navβ4 peptide was 100 μM. **(C,D)** Voltage dependence of the average *I_NaR_* amplitude generated by rpNav1.7 (*n* = 14) and its mutants Y392K (*n* = 11), N395K (*n* = 6), L398K (*n* = 6), V947K (*n* = 9), F1737K (*n* = 9), and Y1744K (*n* = 11). *I_NaR_* was normalized to the peak transient current elicited at −10 mV. **(E)** Summary of the average *I_NaR_* amplitude elicited at −30 mV. ***p < 0.0001.

Navβ4 peptide reduces the inhibition of sodium currents by lidocaine, whose receptor site situates in the pore region of VGSCs (Bant et al., 2013; Gamal El-Din et al., 2018). Therefore, we hypothesized that the receptor site of Navβ4 peptide is also located within channel pore region. Here we focused on six residues (Y392, N395, L398, V947, F1737, and Y1744) that lie within the receptor site region for lidocaine and other local anesthetics (Nau and Wang, 2004; Nguyen et al., 2019; Wu et al., 2023) (Figure 2A). Each of these residues was substituted with a positively charged residue lysine in rpNav1.7. Lysine scanning has previously been successfully used to interrogate the pore residues involved in the actions of local anesthetics on VGSCs (Wright et al., 1998) and we predicted that lysine substitutions in the channels could robustly impact *I_NaR_* generation due to electrostatic repulsion.

**Figure 3:**
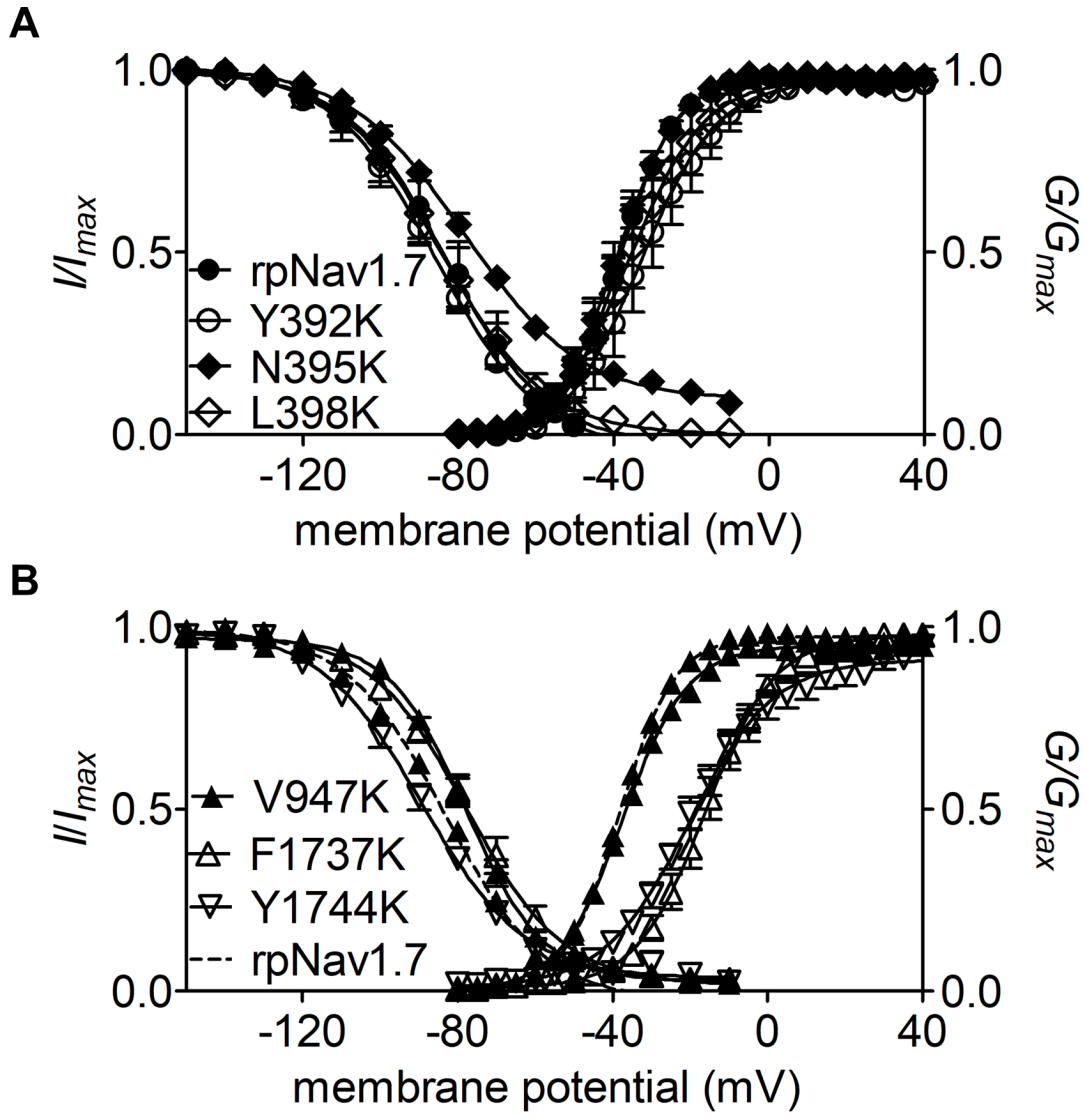
Effects of mutations in rpNav1.7 on steady-state activation and inactivation of the channel in the presence of 100 μM Navβ4 peptide. **(A)** Mutations Y392K, N395K, and L398K in DI-S6. **(B)** Mutations V947K, F1737K, and Y1744K in DII-S6 and DIV-S6. Steady-state activation and inactivation were assayed as described in Figures 1E,F. All curves were fitted to a Boltzmann function and the V_1/2_ values yielded were summarized in Table 1.

The N395K mutation at DI-S6 substantially reduced the capability of rpNav1.7 to generate Navβ4 peptide mediated *I_NaR_* (Figures 2B,C). The average *I_NaR_* amplitude elicited at −30 mV was 2.6% ± 0.5% of the peak transient current, one-sixth of the value observed with rpNav1.7 (*p* < 0.0001). A similar reduction was also observed at other potentials ranging from −55 to −10 mV (Figures 2C,E). However, persistent currents, which arose nearly instantaneously and did not significantly decay within 100 ms, were greatly enhanced. The persistent current measured at −10 mV was 7.1% ± 3.0% (rpNav1.7) and 16.1% ± 4.9% (N395K) of the peak transient current (*p* < 0.0001), respectively. This might be caused by an increase in the “window currents” formed by superimposition of steady-state activation and inactivation curves, which typically result in persistent currents (Attwell et al., 1979). In Figure 3A, the N395K mutation substantially increased the fraction of rpNav1.7 channel that did not inactivate at voltages more positive than −60 mV, although it did not significantly change the V_1/2_ values of voltage dependence of steady-state activation or inactivation (Table 1). Interestingly, in contrast to Navβ4 peptide, under control condition N395K had no effect on steady-state inactivation of rpNav1.7 but shifted channel activation to more negative potentials (Supplementary Figure S1), which are consistent with our previous findings comparing WT Nav1.7 to Nav1.7-N395K (Sheets et al., 2011).

The mutations F1737K and Y1744K at DIV-S6 also greatly reduced the capability of rpNav1.7 to generate Navβ4 peptide mediated *I_NaR_*. While the former caused a complete loss of *I_NaR_*, the latter reduced 57.3% of the *I_NaR_* elicited at −30 mV (Y1744K, 6.7% ± 1.1% *vs* rpNav1.7, *p* < 0.0001; Figures 2D,E). The reduction mainly occurred at voltages ranging from −55 to −20 mV (Figure 2D), and a positive shift of voltage dependence of *I_NaR_* activation was also observed. These two mutations positively shifted voltage dependence of channel activation by 22.6 mV and 19.8 mV, respectively, but none of them significantly changed voltage dependence of steady-state inactivation.

The three other mutations, Y392K, L398K, and V947K, located within DI-S6 and DII-S6 neither changed the average *I_NaR_* amplitude nor shifted voltage dependence of *I_NaR_* activation (Figures 2C,D), suggesting that these residues might not play roles in Nav1.7’s ability to generate Navβ4 mediated *I_NaR_*. These three mutations did not affect steady-state activation or inactivation of rpNav1.7 either (Table 1; Figures 3A,B).

### Identification of the FHF4A sequence mediating *I_NaR_*

**Figure 4:**
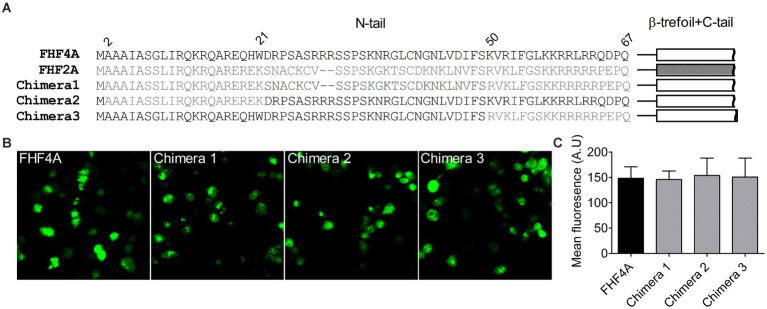
Transplantation of FHF2A N-terminus did not alter the GFP-fusion FHF4A expression in ND7/23 cells. **(A)** Sequence alignment of the N-terminus of FHF4A, FHF2A and three chimera constructs. The numbers above the sequences indicate the positions of residues in FHF4A N-tail. **(B)** Fluorescence showed expression levels of the GFP-fusion FHF4A and three chimeras. **(C)** Summary of fluorescence in ND7/23 cells transiently transfected with the GFP-fusion FHF4A and three chimeras. The data for each group comes from three separate experiments. FHF4A *vs* Chimera 1, *p* > 0.05; *vs* Chimera 2, *p* > 0.05; *vs* Chimera 3, *p* > 0.05.

Next, we investigated if FHF4A, another *I_NaR_* mediator reported recently (White et al., 2019; Xiao et al., 2022), shares the receptor site in VGSCs with Navβ4 peptide. Our previous work showed that a synthetic peptide containing the sequence FHF4A A2 – W21 could reconstitute the Nav1.8 *I_NaR_* mediated by full-length FHF4A (Xiao et al., 2022). However, another study reported that *I_NaR_* was also reconstituted in Purkinje neurons by a peptide derived from the sequence FHF4A K50 – R63, and that substitution of the 7^th^ residue leucine with serine makes the peptide no longer induce *I_NaR_* (White et al., 2019). The residue serine appears at position 54 in FHF2A, an isoform inducing threefold smaller Nav1.8 *I_NaR_* than FHF4A (Xiao et al., 2022). To verify the amino acid segment responsible for *I_NaR_* mediation in intact FHF4A, we constructed three chimeras between FHF4A and FHF2A (Figure 4A). In chimera 1, the entire N-terminus of FHF4A was replaced with that of FHF2A. In chimera 2 and 3, the segments A2 –W21 and K50 – R63 were replaced with those at the corresponding positions in FHF2A, respectively. All constructs had a GFP fluorescent tag fused to them. When transiently transfected into ND7/23 cells, the mean GFP fluorescence intensity remained unchanged, suggesting that none of three replacements significantly changed FHF4A expression level in heterologous expression system (Figures 4B,C; *N* = 3, *p* > 0.05, one-way ANOVA with *post hoc* Tukey test).

**Figure 5:**
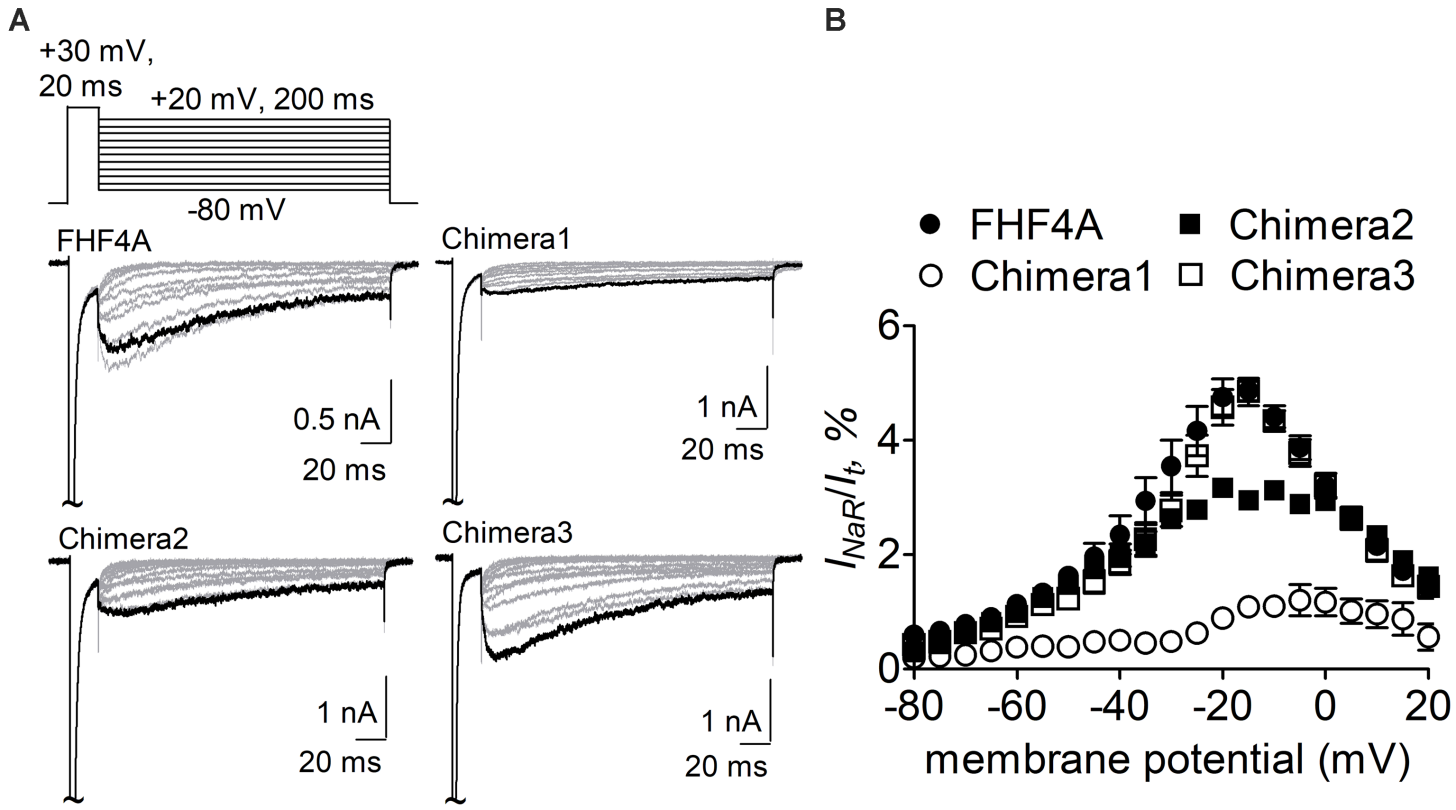
Transplantation of FHF2A N-terminus reduced the capability of FHF4A to generate Nav1.8 *I_NaR_* in ND7/23 cells. **(A)** Families of typical Nav1.8 *I_NaR_* traces mediated by FHF4A and three chimeras. *I_NaR_* were induced by a standard *I_NaR_* protocol (*inset*), in which cells were applied by 200-ms repolarizing voltage steps from +20 to −80 mV in −10 mV increments, following a 20-ms depolarizing potential of +30 mV. Cells were held at −100 mV. **(B)** Voltage dependence of average Nav1.8 *I_NaR_* mediated by FHF4A (*n* = 10), chimera 1 (*n* = 8), chimera 2 (*n* = 5) and chimera 3 (*n* = 10). *I_NaR_* was normalized to the peak transient current elicited at 0 mV.

The Nav1.8 construct was then transiently co-transfected with WT FHF4A or the chimeras into ND7/23 cells. As reported recently, WT FHF4A induced large Nav1.8 *I_NaR_*, which displayed slow onset and decay kinetics, peaked at −15 mV (Figure 5A), and was measured to be 4.9% ± 0.2% of the peak transient current (Figure 5B). Compared with WT FHF4A, transplantation of the entire FHF2A N-terminus into FHF4A resulted in a threefold decrease Nav1.8 *I_NaR_* (chimera 1, 1.1% ± 0.1% *vs* WT, *p* < 0.0001), indicating that the amino acid segment responsible for FHF4A to mediate *I_NaR_* is located within the N-terminus of FHF4A. Transplantation of FHF2A A2-W21 approximately 1-fold reduced the average *I_NaR_* amplitude (chimera 2, 2.9% ± 2.0% *vs* WT, *p* < 0.0001). However, surprisingly, transplantation of FHF2A R48 – R61 failed to change the average amplitude (chimera 3, 4.8% ± 0.2% *vs* WT, *p* > 0.05). Moreover, none of three chimeras evidently shifted voltage dependence of activation of Nav1.8 *I_NaR_* (Figure 5B). These results strongly suggest that the segment critical for full-length FHF4A to mediate the *I_NaR_* generated by VGSCs, at least in Nav1.8, is located at the very beginning, not at the end, of the N-terminus. Accordingly, the synthetic 20-residue peptide AAAIASGLIRQKRQAREQHW was used to further interrogate the receptor site of FHF4A in Nav1.8 pore region.

**Figure 6:**
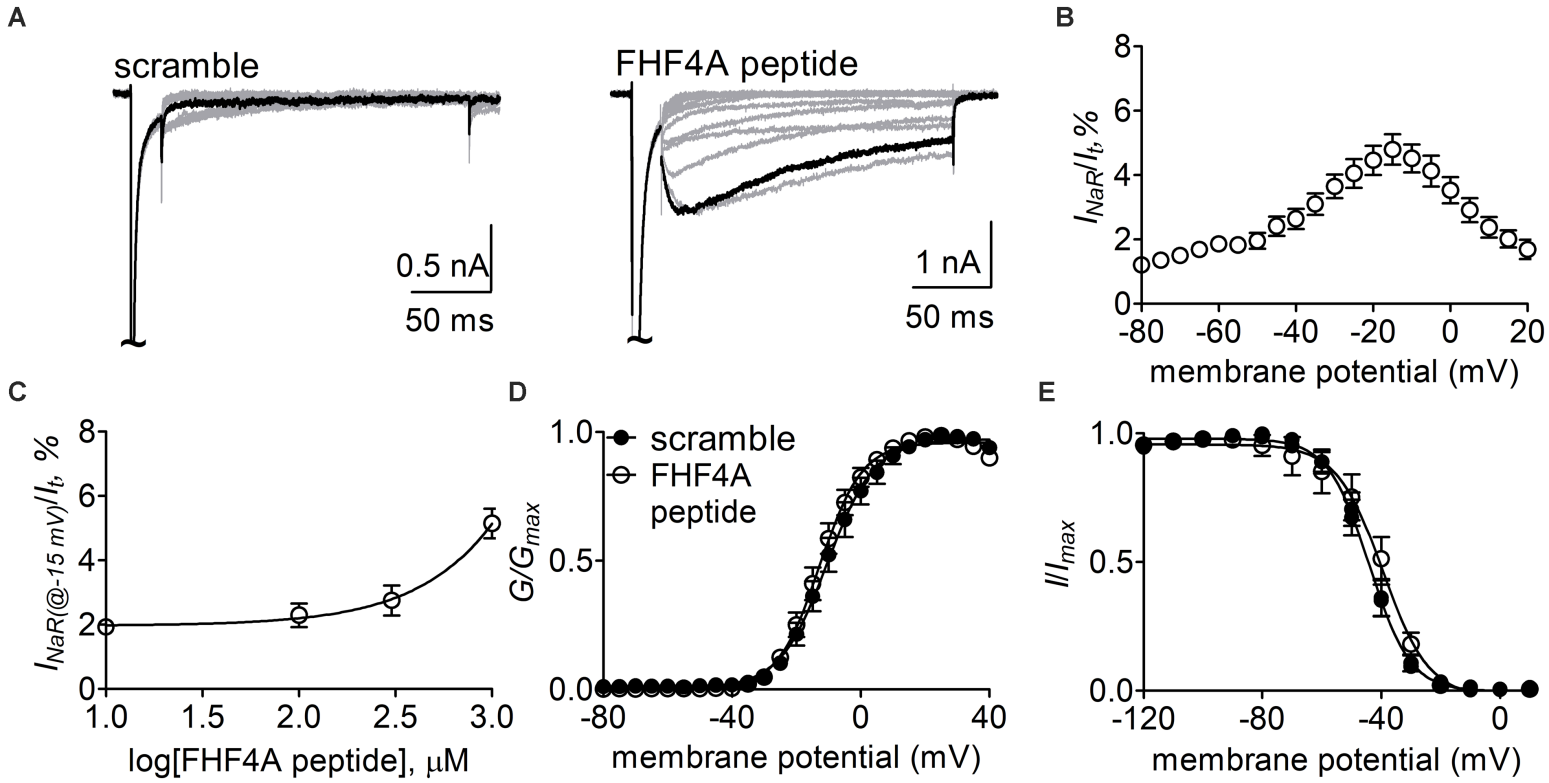
FHF4A peptide mediated *I_NaR_* generated by Nav1.8 co-expressed with FHF2B in ND7/23 cells. **(A)** Typical current traces elicited by the protocol shown in Figure 5A (*inset*) in the presence of 1 mM scrambled peptide or FHF4A peptide. **(B)** Voltage dependence of the average Nav1.8 *I_NaR_* mediated by 1 mM FHF4A peptide (*n* = 9). *I_NaR_* were normalized to the transient peak current evoked at 0 mV. **(C)** Concentration-dependent curve of FHF4A peptide inducing Nav1.8 *I_NaR_* at −15 mV. Each data point comes from 5 to 9 separated cells. **(D,E)** Effects of FHF4A peptide on steady-state activation and inactivation of Nav1.8. Families of sodium currents were induced by 50-ms depolarizing steps to various potentials ranging from −80 to +40 mV in 5-mV increments. Steady-state inactivation was estimated using a standard double pulse protocol in which sodium currents were induced by a 50-ms depolarizing potential of 0 mV following a 500-ms prepulse at potentials that ranged from −100 to +10 mV with a 10-mV increment. Cells were held at −100 mV. All curves were fitted to a Boltzmann function. V_1/2 (activation)_: scramble, −10.0 ± 2.4 mV (*n* = 8) *vs* FHF4A peptide, −12.5 ± 1.7 (*n* = 10), *p* > 0.05. V_1/2 (inactivation)_: scramble, −44.5 ± 2.1 mV (*n* = 8) *vs* FHF4A peptide, −42.4 ± 4.3 (*n* = 10), *p* > 0.05.

Next, the Nav1.8 construct was co-transfected with FHF2B, a FHF isoform not capable of mediating *I_NaR_* generation but previously shown to increase Nav1.8 expression level, into ND7/23 cells. FHF4A peptide (also known as F4A, Xiao et al., 2022) at desired concentrations was applied into the intracellular solution. In Figure 6A, while Nav1.8 failed to produce *I_NaR_* in the presence of 1 mM scrambled protein, the Nav1.8 *I_NaR_* shown in Figure 5A were fully reconstituted in the presence of 1 mM FHF4A peptide. FHF4A peptide mediated *I_NaR_* was observed at voltages ranging from −40 to +20 mV, peaked at −15 mV (Figure 6B), and was measured to be 4.8% ± 0.5% of the peak transient current. FHF4A peptide mediated *I_NaR_* fluxing through Nav1.8 in a concentration-dependent manner. According to the concentration-dependent curve, the effect of the peptide did not saturate at 1 mM, and fitting the Hill equation to the data roughly yielded an EC50 value of 20.04 mM (Figure 6C). In contrast to Navβ4 peptide, FHF4A peptide did not alter steady-state activation or inactivation (Figures 6D,E).

### Effects of the residues in Nav1.8 pore region on FHF4A peptide *I_NaR_*

**Figure 7:**
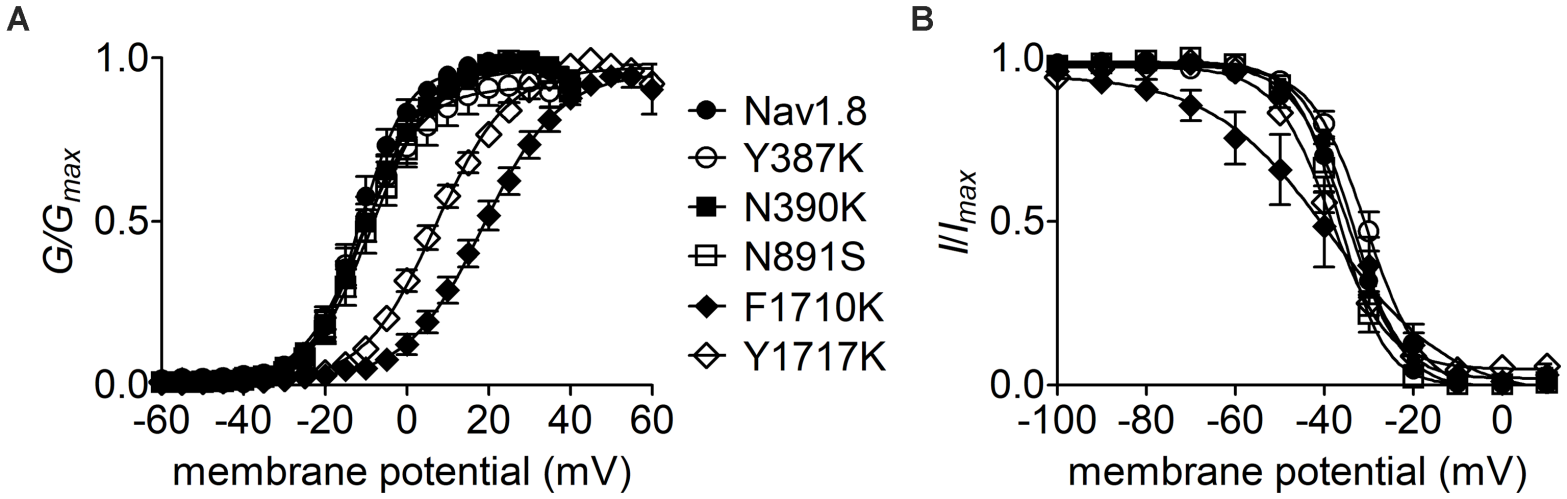
Effects of mutations in Nav1.8 on steady-state activation **(A)** and inactivation **(B)** of the channel in ND7/23 cells co-transfected by FHF2B. Families of sodium currents were evoked as described in Figures 6D,E. FHF4A peptide (1 mM) was applied in the intracellular solution. All curves were fitted to a Boltzmann function and the V_1/2_ values yielded were summarized in Table 2.

The Y392, N395, F1737, and Y1744 in Nav1.7 are highly conserved in VGSC subtypes and correspond to Y387, N390, F1710, and Y1717 in Nav1.8, respectively. As observed for Nav1.7, lysine-substitution of F1710 and Y1717, but not Y387K nor N390K, positively shifted voltage dependence of activation of Nav1.8, by 31.0 mV and 18.4 mV, respectively, (Figure 7A; Table 2). None of four lysine-mutations significantly changed the V_1/2_ value of steady-state inactivation (Figure 7B; Table 2).

**Table 2:** Gating properties of Nav1.8 and the mutants in the presence of 1 mM FHF4A peptide.

	Nav1.8	Y387K	N390K	F1710K	Y1717K	N891S
Activation (V_1/2_, mV)	−11.1 ± 1.7 (9)	−9.9 ± 2.1 (10)	−9.7 ± 1.8 (7)	19.9 ± 2.0*** (7)	7.3 ± 1.5*** (10)	−8.6 ± 2.1 (6)
Inactivation (V_1/2_, mV)	−34.2 ± 1.8 (9)	−30.7 ± 1.5 (10)	−33.5 ± 1.0 (7)	−41.1 ± 5.5 (7)	−38.4 ± 1.0 (10)	−36.4 ± 0.7 (6)
*I_NaP_*, %	3.0 ± 1.8 (9)	3.6 ± 0.9 (8)	5.6 ± 2.2^*^ (7)	0.8 ± 0.9^**^ (6)	5.4 ± 3.8 (10)	1.1 ± 0.5^***^ (6)

Midpoint voltages of the steady-state activation and inactivation curves in Figure 7 were determined with a standard Boltzmann distribution fit. *I_NaP_* measured at the end of the 50-ms pulse of +10 mV was normalized to the peak transient current. The number of separate cells tested is indicated in parentheses. *p < 0.05; **p < 0.01; ***p < 0.0001 *vs* Nav1.8.

**Figure 8:**
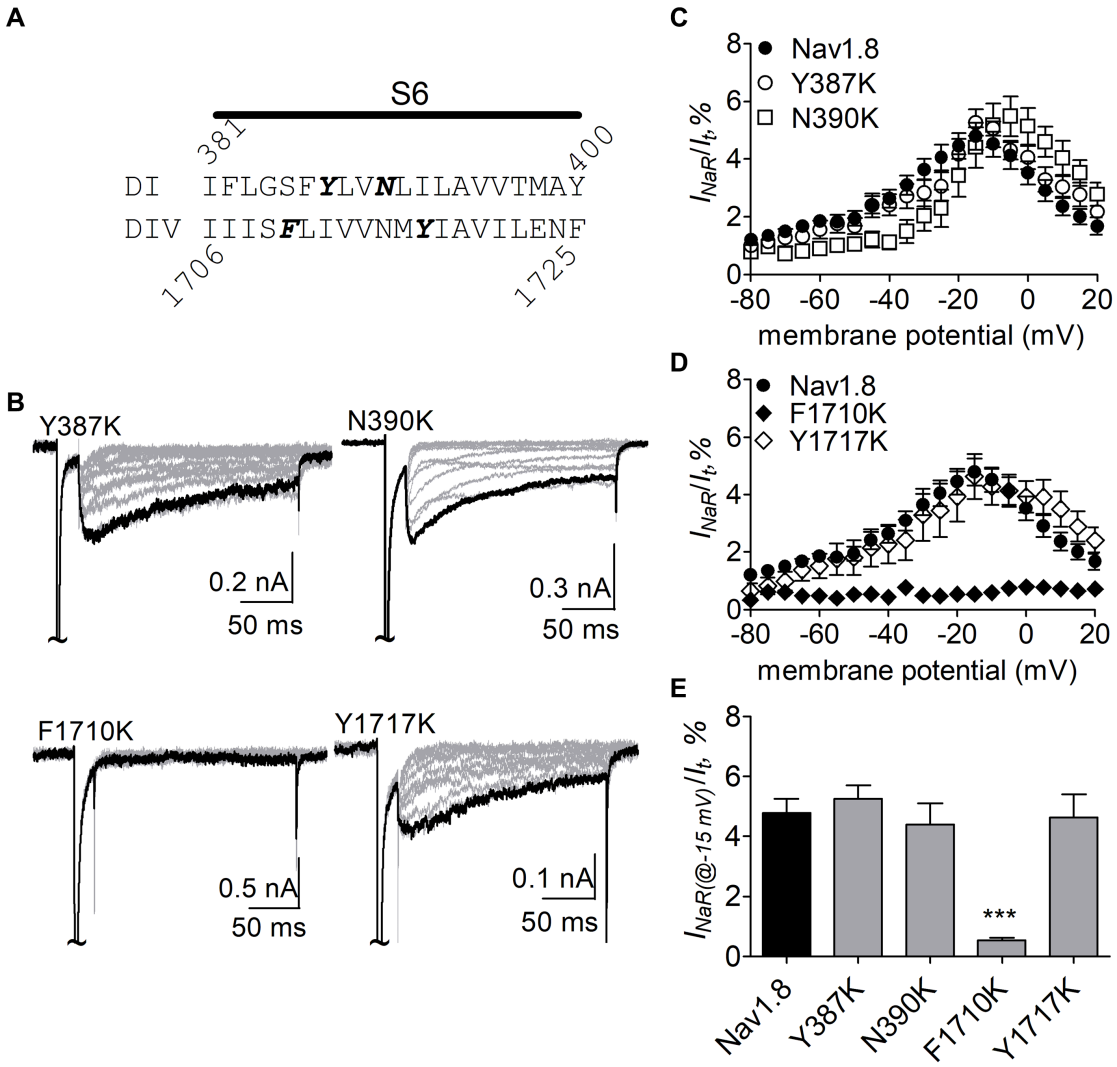
Effects of mutations in Nav1.8 on FHF4A peptide *I_NaR_* in ND7/23 cell co-transfected by FHF2B. **(A)** Amino acid sequence of Nav1.8 DI-S6 and DIV-S6. The residues of interest are highlighted in bold. **(B)** Typical Nav1.8 *I_NaR_* traces elicited by the *I_NaR_* protocol shown in Figure 5A (*inset*). **(C,D)** Voltage dependence of average *I_NaR_* generated by the Nav1.8 mutants Y387K (*n* = 8), N390K (*n* = 7), F1710K (*n* = 6), and Y1717K (*n* = 10). **(E)** Summary of *I_NaR_* at −15 mV. In **(C–E)** *I_NaR_* was normalized to the peak transient current evoked at 0 mV. 1 mM FHF4A peptide was applied in the intracellular solution. ***p < 0.0001.

The F1710K mutation rendered Nav1.8 resistant to FHF4A peptide mediated *I_NaR_*. In the presence of 1 mM FHF4A peptide, the F1710K mutation caused a ninefold decrease of the average *I_NaR_* amplitude elicited at −15 mV (F1710K, 0.5% ± 0.1%, *n* = 6 *vs* Nav1.8, *p* < 0.0001; Figures 8A–E). The N390K mutation slightly shifted the voltage dependence of Nav1.8 *I_NaR_* to more positive potentials, but it did not significantly change the average *I_NaR_* amplitude (N390K, 4.4% ± 0.7% *vs* Nav1.8, *p* > 0.05; Figures 8C,E). The other two mutations Y387K and Y1717K neither affected the current–voltage relationship of Nav1.8 *I_NaR_* nor significantly changed the average *I_NaR_* amplitude (Y387K, 5.3% ± 0.5% *vs* Nav1.8, *p* > 0.05; Y1717K, 4.6% ± 0.8% *vs* Nav1.8, *p* > 0.05; Figure 8E). These data suggest that the residue F1710, but not Y387, N390, or Y1717, plays a critical role in Nav1.8 producing FHF4A peptide *I_NaR_*.

### N891 unique in neuronal TTX-resistant VGSCs is critical for Nav1.8 sensitive to FHF4A peptide

**Figure 9:**
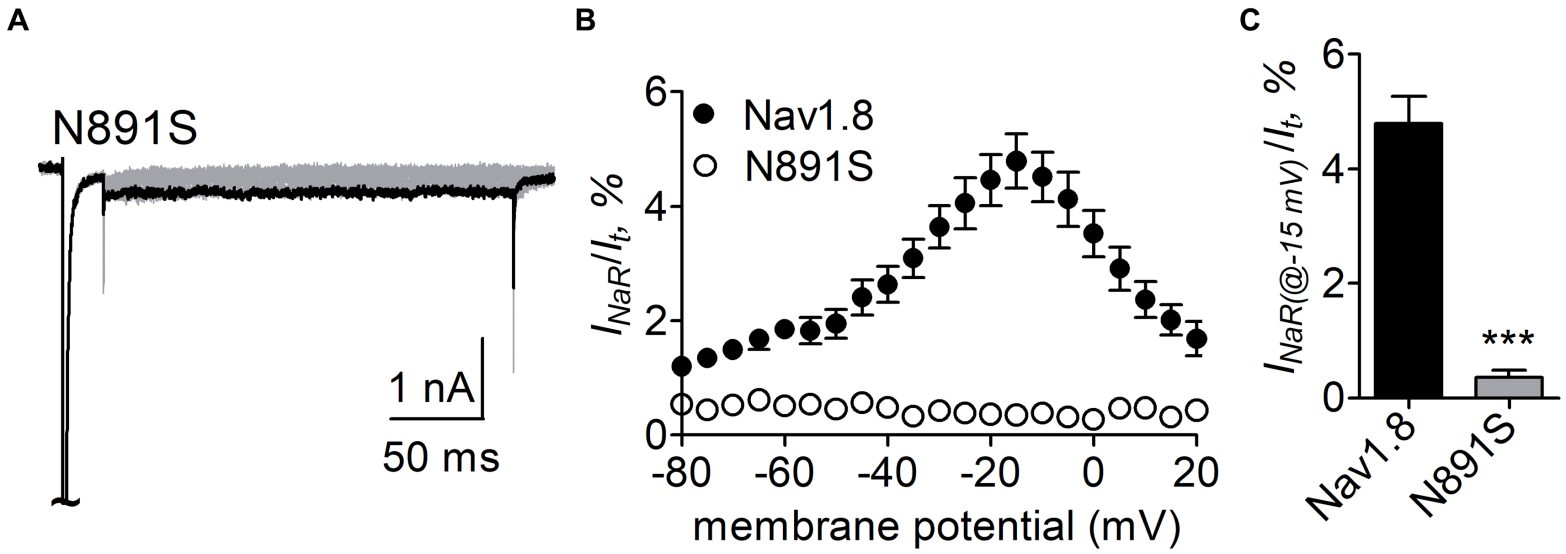
N891S abolished FHF4A peptide *I_NaR_* in Nav1.8 co-expressed with FHF2B in ND7/23 cells. **(A)** Typical *I_NaR_* traces generated by the Nav1.8 N891S mutant. *I_NaR_* was elicited by the *I_NaR_* protocol shown in Figure 5A (*inset*). The current trace elicited at −15 mV was highlighted. **(B)** Voltage dependence of average *I_NaR_* generated by Nav1.8 (*n* = 9) and N891S (*n* = 6). **(C)** Summary of *I_NaR_* at −15 mV. *I_NaR_* was normalized to the peak transient current evoked at 0 mV. 1 mM FHF4A peptide was applied in the intracellular solution. ***p < 0.0001.

N891 at the end of DII-S6 is unique in two neuronal TTX-resistant VGSCs Nav1.8 and Nav1.9. The residue is replaced with a serine at the corresponding position in other VGSC subtypes. The N891S mutation had no effect on steady-state activation or inactivation of Nav1.8 (Figure 7; Table 2), but completely abolished the capability of the channel to generate FHF4A peptide mediated *I_NaR_*. In the presence of 1 mM FHF4A peptide, no *I_NaR_* was induced by repolarizing potentials ranging from −80 to +20 mV (Figures 9A–C).

### Role of N891 and F1710 in Nav1.8 generating full-length FHF4A *I_NaR_*

**Figure 10:**
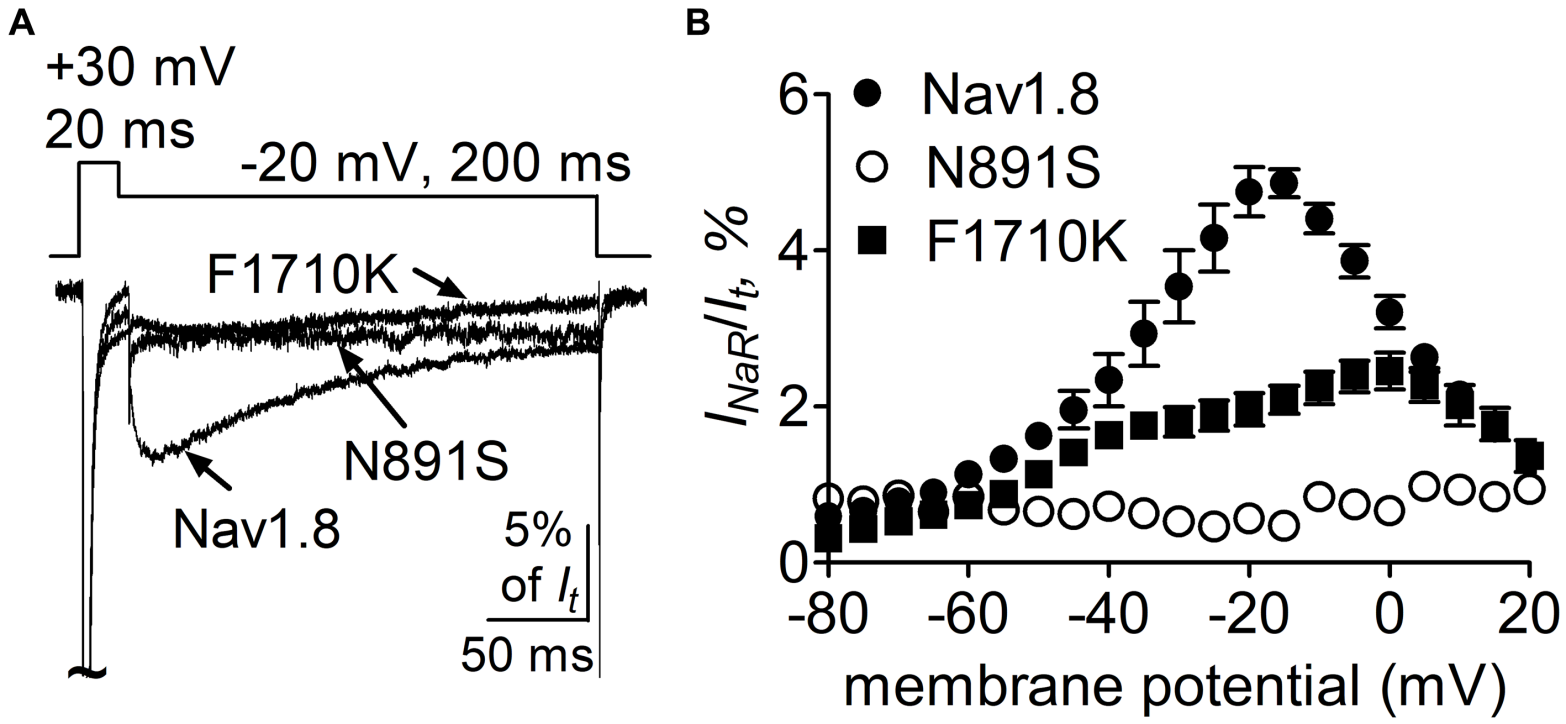
Mutations N891S and F1710K decreased the capability of Nav1.8 to generate full-length FHF4A *I_NaR_* in ND7/23 cells. **(A)** Superimposed current traces of FHF4A-mediated *I_NaR_* elicited by a 200-ms repolarizing potential of −20 mV following a 20-ms depolarizing potential of +30 mV. **(B)** Voltage dependence of the average FHF4A-mediated *I_NaR_* generated by Nav1.8 (*n* = 10), N891S (*n* = 5) and F1710K (*n* = 7). *I_NaR_* were normalized to the peak transient current elicited at 0 mV.

We further confirmed the roles of the above-identified critical residues in *I_NaR_* mediated by full-length FHF4A, but not by full-length Navβ4, because full-length Navβ4 fails to reconstitute *I*_NaR_ in heterologous systems when co-expressed with VGSC α-subunits. In Figure 10A, co-expression of full-length FHF4A with the N891S channel did not generate *I*_NaR_ in ND7/23 cells. FHF4A could still induce *I_NaR_* from the F1710K channel (Figure 10A), but the average *I_NaR_* amplitude at −20 mV is only 2.0% ± 0.2% of the peak transient current, which is less than one-half of the value from WT Nav1.8 (*p* < 0.0001). The reduction was also observed at voltages ranging from −35 mV to 0 mV (Figure 10B).

### FHF4A peptide inhibited Navβ4 peptide-mediated *I_NaR_* in rpNav1.7

**Figure 11:**
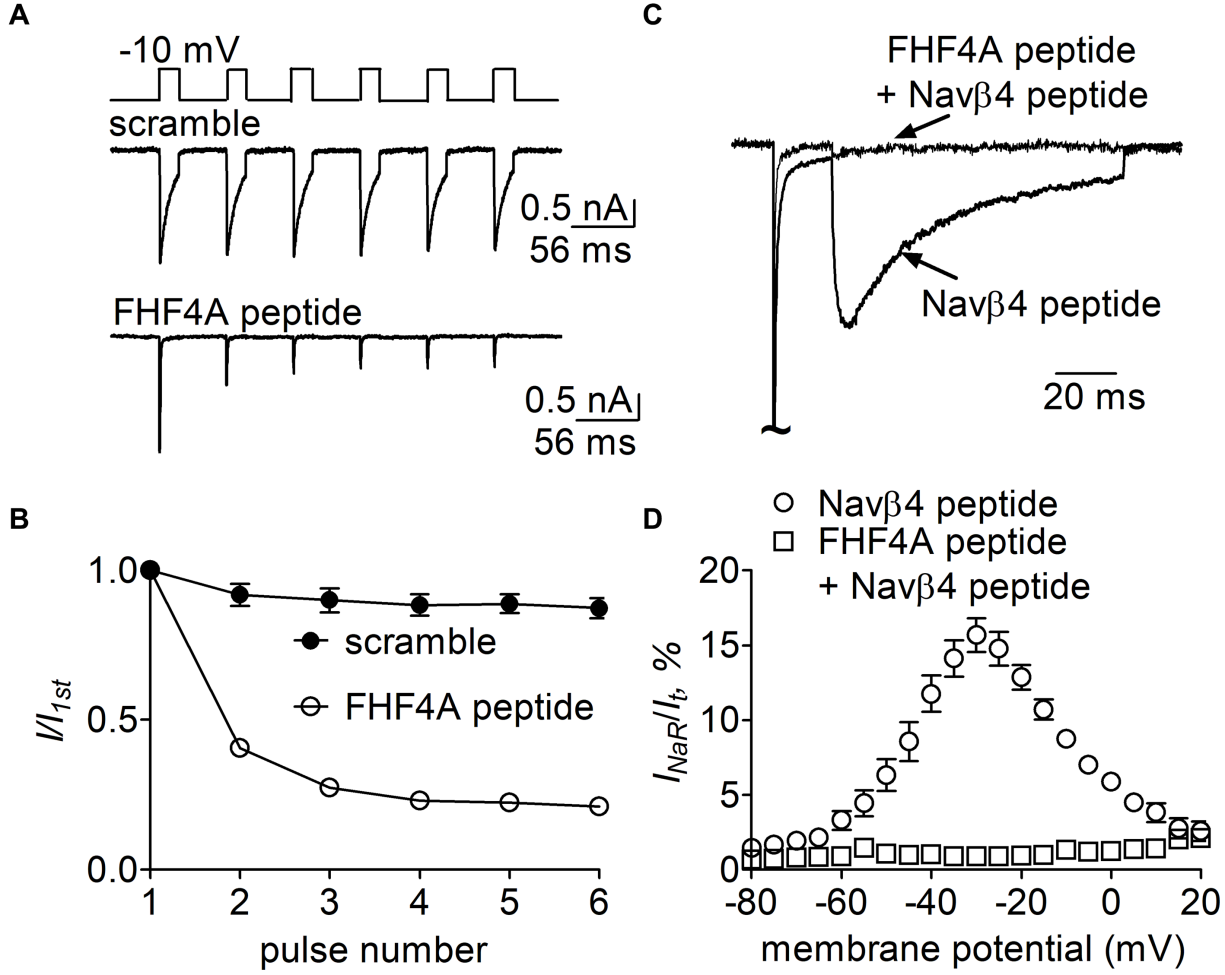
Effects of FHF4A peptide on Navβ4 peptide-mediated rpNav1.7 *I_NaR_* in HEK293 cells. **(A)** Typical current traces of long-term inactivation in the presence of 1 mM scramble (*n* = 6) or FHF4A peptide alone (*n* = 5). The current was elicited by six 16-ms depolarization pulses of −10 mV from a holding potential of −100 mV with −100 mV 40-ms interpulse recovery phases beween each depolarization pulse (*inset*). **(B)** Effect of FHF4A peptide on long-term inactivation of rpNav1.7. **(C)** Superimposed *I_NaR_* traces in the presence of 100 μM Navβ4 peptide alone or 100 μM Navβ4 peptide +1 mM FHF4 peptide. Currents were elicited by a 100-ms repolarizing potential of −30 mV following a 20-ms depolarzing potential of +30 mV. **(D)** Voltage dependence of the average *I_NaR_* in the presence of Navβ4 peptide alone (*n* = 14) or Navβ4 peptide + FHF4 peptide (*n* = 5).

Finally, we examined whether FHF4A peptide competed with Navβ4 peptide in VGSCs. In Figure 11A, 1 mM FHF4A peptide decreased the fraction of the current elicited at the 6th pulse, from 0.87 ± 0.08 (scramble) to 0.21 ± 0.03 (*p* < 0.0001; Figure 11B), indicating that FHF4A peptide interacted with rpNav1.7 and evidently enhanced accumulation of long-term inactivation of rpNav1.7. Our previous work showed that co-expression of full-length FHF4A did not induce Nav1.7 *I_NaR_* in heterologous system (Xiao et al., 2022). In the presence of FHF4A peptide, the rpNav1.7 *I_NaR_* mediated by Navβ4 peptide was inhibited completely (Figures 11C,D). These results suggest that FHF4A peptide competes with Navβ4 peptide for binding and action on VGSCs.

## Discussion

We investigated the interaction between the Navβ4 peptide, A-type FHFs and VGSCs. Our data identifies specific inner pore residues of Nav1.7 and Nav1.8 that interact with the Navβ4 peptide and A-type FHFs. While both the Navβ4 peptide and N-terminus of the A-type FHFs can act as pore blockers, they are not identical in their interactions with the VGSC pore residues. Furthermore, the A-type FHF isoforms can have distinct effects on VGSCs. Understanding the molecular determinants of these interactions may aid the development of novel sodium channel modulators.

A-type FHFs have four isoforms FHF1A – FHF4A. They consist of a long N-terminus, an FGF-like β-trefoil core, and a short C-terminus (Goldfarb, 2005). They display an intrinsic capability to generate *I_NaR_*, to a varying extent, in Nav1.8 and Nav1.9. FHF4A can induce threefold larger *I*_NaR_ from Nav1.8 than FHF2A (Xiao et al., 2022). In this study, we show that the distinct capability results from divergent N-terminus because replacement with the whole FHF2A N-terminus (chimera 1) exactly threefold decreased FHF4A generated *I_NaR_*. Our chimera data also show that in A-type FHFs the amino acid segment responsible for *I_NaR_* generation mainly situates at the very beginning, but not at the end, of their N-terminus. Firstly, replacement with FHF2A A2-K21 (chimera 2) onefold decreased FHF4A generated *I_NaR_*. Secondly, a short peptide derived from this segment can fully reconstitute the Nav1.8 *I_NaR_* induced by intact FHF4A. Thirdly, replacement of K50-R63 with the corresponding part in FHF2A (chimera 3) neither modified voltage dependence of activation nor changed the average amplitude of FHF4A-mediated Nav1.8 *I_NaR_*. The segment K50-R63 is adjacent to the β-trefoil core and exhibits high sequence similarity to Navβ4 peptide. Although a short peptide derived from this segment has been shown to induce robust Nav1.6 *I_NaR_* in Purkinje neurons (White et al., 2019), co-expression of either full-length FHF4A or FHF2A with Nav1.6 fails to generate *I_NaR_* in heterologous system (Xiao et al., 2022). However, we cannot rule out the possibility that either FHF4A or Nav1.6 are post-translationally modified in neurons in a way that allows the combination to induce *I_NaR_* in neurons. Our data also indicate that some of the residues located within D22-S49 can also contribute to FHF4A *I_NaR_* generation, at least in Nav1.8, because the *I_NaR_* induced by chimera 1 was nearly twofold smaller than the *I_NaR_* induced by chimera 2.

Our present data demonstrate that *I_NaR_* mediators partially share the receptor site with local anesthetics in VGSCs. The receptor site of local anesthetics is formed by multiple residues within DIS6, DIIIS6 and DIVS6. We have previously reported that the N395K mutation attenuates the inhibitory effects of lidocaine on Nav1.7 (Sheets et al., 2007). Several studies have implicated F1764 and Y1771 in Nav1.2 (corresponding to F1737 and Y1744 in Nav1.7) as playing a major effect on local anesthetics-induced inhibition of VGSCs (Ragsdale et al., 1996). We recently reported that mutation of an asparagine within DII-S6, N945, could render Nav1.7 resistant to Navβ4 peptide (Xiao et al., 2022). Taken together with this result, our present data indicate that the receptor site of Navβ4 peptide consists of at least four residues (N395, N945, F1737, and Y1744) in Nav1.7, and the first three display a profound impact on the capability of Nav1.7 to produce Navβ4 peptide *I_NaR_*. One potential limitation is that the receptor site is determined from Nav1.7-R1559P, not from WT Nav1.7. R1599P mutant channels were used here as they generate larger *I_NaR_* than WT channels in HEK293 cells. Inflammatory mediators enhance TTX-sensitive *I_NaR_* in DRG neurons (Tan et al., 2014), as do multiple PEPD mutations in Nav1.7 (Theile et al., 2011). We used the R1599P mutation because, as with PEPD mutations, it impairs Nav1.7 inactivation but is located at the outer part of DIV-S4, where the mutation is less likely than many of the PEPD mutations to directly alter the arrangement of the channel pore. Consistent with our findings, Wang et al. (2006) demonstrated that the phenylalanine corresponding to Nav1.7-F1737 played a critical role in WT Nav1.5 to generate Navβ4 peptide *I_NaR_*. These residues are highly conserved in Nav1.1 – Nav1.8 that are sensitive to Navβ4 peptide. Among the four residues, only a phenylalanine is involved in formation of receptor site of FHF4A peptide in Nav1.8. F1710K, but not N390K or Y1717K, significantly reduced Nav1.8 *I_NaR_* mediated by FHF4A peptide. Mutating the asparagine that corresponds to N945 in Nav1.7 does not affect Nav1.5 and Nav1.9 ability to produce FHF2A *I_NaR_* (Xiao et al., 2022), indicating this residue is situated out of the receptor site for the FHF4A peptide.

**Figure 12:**
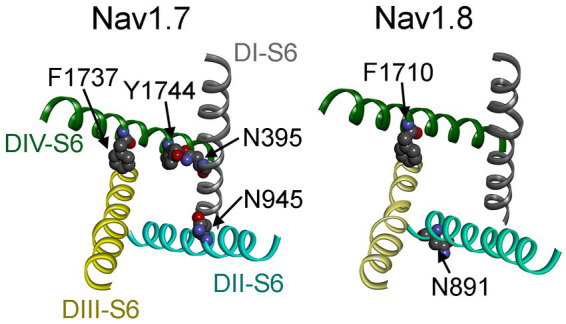
Channel pore structures of Nav1.7 (*left*) and Nav1.8 (*right*). The structures composed of the S6 segments of four domains (DI–DIV) are derived from the cryo-electron microscopy structures of human Nav1.7 (PDB ID: 6J8I) and human Nav1.8 (PDB ID: 7WE4). The top view illustrates that the side chains of the residues of interest are closely associated with the inner pore of VGSCs.

Our data also indicate that these two distinct *I_NaR_* mediators only partially share the receptor site with each other. While the asparagine at DII-S6, N954 (Nav1.7 numbering), is critical for VGSC sensitivity to Navβ4 peptide, a different asparagine in the same transmembrane segment of Nav1.8, N891 (Nav1.8 numbering), is a major determinant of FHF4A *I_NaR_* in Nav1.8. N891 emerges only in Nav1.8 and Nav1.9 and is substituted by a serine in Nav1.1 – Nav1.7. As the N891S mutation rendered Nav1.8 resistant to either full-length FHF4A or FHF4A peptide, this serine may potentially explain why the VGSC subtypes Nav1.5, Nav1.6 and Nav1.7 failed to generate FHF4A *I_NaR_* in our previous work. Moreover, we predict that N891 is not involved in Navβ4 peptide interaction with VGSCs because the VGSC subtypes (e.g., Nav1.6 and Nav1.8) carrying a serine or an asparagine at this position are both reported to be able to generate Navβ4 peptide *I_NaR_* (Patel et al., 2016; Tan et al., 2014). N891 is the eighth residue downstream of the asparagine crucial for VGSCs sensitivity to Navβ4 peptide, suggesting that Navβ4 peptide may bind more deeply in VGSC’s inner pore than FHF4A peptide (Figure 12).

Partial overlapping of the receptor sites supports the idea that Navβ4 peptide and FHF4A peptide mediate *I_NaR_* generation through a similar relief-of-open-channel-block mechanism. This mechanism requires that the peptides compete with the fast inactivation particle to block open VGSCs at strong positive potentials and unblocks upon repolarization so that the channels can reconduct (Cannon and Bean, 2010). Indeed, like Navβ4 peptide, FHF4A peptide accelerates fast inactivation after Nav1.8 opens (Xiao et al., 2022). Moreover, cryo-EM structures of Nav1.7 and Nav1.8 have clearly revealed that the side chains of the key residues that we identified within the receptor sites, except for N891, project into channel inner pore (Figure 12; Shen et al., 2019; Huang et al., 2022). These findings strongly indicate that both peptides can function as open-channel blockers. Our mutagenesis data further indicate that the peptides bind to channel inner pore mainly by hydrophobic and hydrophilic interactions, but not ionic interactions. Although both positive and hydrophobic residues in Navβ4 peptide and FHF2A peptide (another *I_NaR_* mediator derived from FHF2A N-terminus) are reported to be essential for inducing *I*_NaR_ (Lewis and Raman, 2011; Xiao et al., 2022), the positive residues seem unlikely to be directly involved in electrostatic salt-bridge interactions with VGSCs because few negative residues are located within VGSC inner pore region. The positive charges carried by Navβ4 peptide and FHF4A peptide are more likely acted on by the electric field across membrane or sensing the driving force for sodium ions, provide the driving force to repel and unbind them from their receptor sites during repolarization.

Our data further demonstrate that FHF2B may facilitate *I_NaR_* generation in VGSCs, although it lacks the ability to directly mediate *I_NaR_*. FHF2B doubles FHF4A peptide mediated Nav1.8 *I_NaR_* in heterologous expression systems (control: 2.5% ± 0.4%, *n* = 6 *vs* FHF2B, 4.8% ± 0.5%, *n* = 9, *p* < 0.05; shown as percent of peak transient current) (Xiao et al., 2022). Increased *I_NaR_* may result from destabilizing fast inactivation or increasing “window currents” of VGSCs (Xiao et al., 2019). FHF2B did not slow Nav1.8 inactivation (τ_fast (@ + 30 mV)_: control, 2.4 ± 0.4 ms, *n* = 8 *vs* FHF2B, 1.0 ± 0.1 ms, *n* = 8, *p* < 0.01; τ_slow (@ + 30 mV)_: control, 13.7 ± 1.7 ms *vs* FHF2B, 11.1 ± 1.7 ms, *p* > 0.05), but it substantially increases “window currents” by negatively shifting channel activation and positively shifting steady-state inactivation (Xiao et al., 2022). Nav1.6 is the major carrier of TTX-sensitive *I_NaR_* in DRG neurons (Cummins et al., 2005; Barbosa et al., 2017). FHF2B significantly slows fast inactivation of Nav1.6 (τ_@ + 30 mV_: control, 0.61 ± 0.03 ms, *n* = 5 *vs* FHF2B, 0.73 ± 0.03 ms, *n* = 5, *p* < 0.05). Consistent with this finding, our previous work has shown that FHF2B overexpression can enhance *I_NaR_* generated by recombinant Nav1.6 in DRG neurons (Barbosa et al., 2017). Since FHF2B docks at the VGSC C-terminus and this FHF core binding site is highly conserved across all VGSC subtypes, we further assume that the molecular manipulation of both TTX-sensitive and TTX-resistant *I_NaR_* can be achieved by either FHF2B knockout or inhibition of FHF2B interaction with VGSC C-terminus in primary neurons.

The role of Navβ4 in *I_NaR_* generation remains controversial. Although the Navβ4 peptide generates robust *I_NaR_*, co-expression of Navβ4 with VGSCs in recombinant systems has never been shown induce *I_NaR_* in heterologous expression systems. Bant and Raman (2010) reported that siRNA induced knock-down of Navβ4 reduced *I_NaR_* generation in cultured cerebellar granular neurons by roughly 60%. However, *in vivo* application of Navβ4 siRNA only reduced *I_NaR_* in DRG neurons by 31% (Xie et al., 2016). Furthermore, *Scn4b* knock-out in mice has produced variable effects on *I_NaR_*. In striatal neurons, *Scn4b* knock-out reduced *I_NaR_* by approximately 67%. In cerebellar Purkinje neurons Ransdell et al. (2017) reported that *Scn4b* knock-out reduced *I_NaR_* by 43% and White et al. (2019) reported that *Scn4b* knock-out had little to no impact on *I_NaR_* amplitudes. Overall, these studies suggest that while Navβ4 may play a role in *I_NaR_* generation in some neurons, other mechanisms for generating *I_NaR_* exist. Further research may be necessary to fully clarify the role of Navβ4 in regulation of VGSCs.

We have shown here that the *I_NaR_* mediator Navβ4 peptide influences gating properties of VGSCs. The peptide shifted voltage dependence of channel activation and inactivation to hyperpolarized potentials. Enhancing Nav1.7 activation can lead to hyperexcitability of DRG neurons (Cummins et al., 2004; Harty et al., 2006), suggesting that Navβ4 peptide regulates neuronal excitability not only by mediating *I_NaR_* but also by modifying gating properties of VGSCs. We also show that Navβ4 peptide displays the capability to modify the effects of painful disorder-associated mutations on Nav1.7 gating properties. The N395K is a hereditary erythromelalgia mutation located within DI-S6 (Drenth et al., 2005). In the absence of Navβ4 peptide, the N395K mutation caused a hyperpolarizing shift of channel activation but failed to alter steady-state inactivation, which is consistent with the observations in our previous work in heterologous expression system (Sheets et al., 2007). However, in the presence of Navβ4 peptide, the N395K mutation did not alter channel activation but shifted steady-state inactivation to depolarized potentials. As Navβ4 is highly expressed in the majority of DRG neurons, it seems likely that the N395K mutation mainly impairs Nav1.7 channel inactivation in primary sensory neurons.

Overall, in this study we extensively investigated the molecular determinants of *I_NaR_* mediated by Navβ4 peptide and FHF4A peptide. We provide evidence that the receptor sites of two *I_NaR_* mediators situate within VGSC inner pore. We show that mutating a phenylalanine that is conserved in Nav1.7 and Nav1.8 renders these VGSCs resistant to Navβ4 peptide and FHF4A peptide, respectively. We also show that FHF2B is capable to enhance *I_NaR_* generation. These findings not only increase understanding of the molecular mechanisms underlying *I_NaR_* generation but also highlight VGSC inner pore region and FHF2B binding site as hotspots to design novel agents targeting *I_NaR_*.

## Data Availability

The raw data supporting the conclusions of this article will be made available by the authors, without undue reservation.
