# Supplementary material for: Molecular determinants of resurgent sodium currents mediated by Navβ4 peptide and A-type FHFs
Source: Front Mol Neurosci. 2024 Oct 2;17:1433981. doi: 10.3389/fnmol.2024.1433981 (PMC11480954; doi:10.3389/fnmol.2024.1433981)
Supplement: Supplementary file 1 [file Data_Sheet_1.pdf]

Supplemental Data

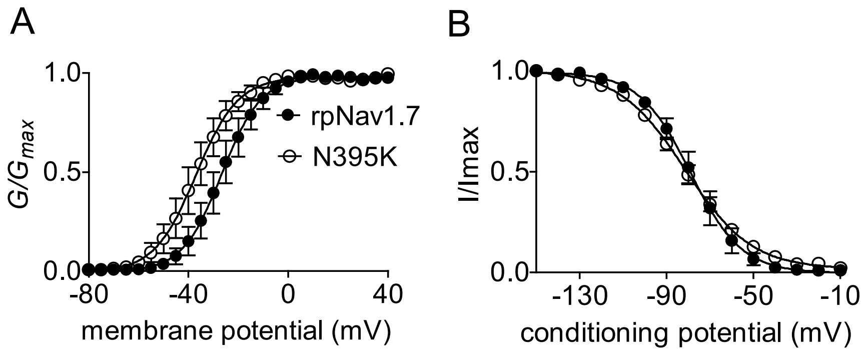

Supplemental Figure 1 Effects of N395K on rpNav1.7 gating properties in the absence of Navβ4 peptide. A, N395K shifted steady-state activation to more negative potentials. Families of sodium currents were induced by 50-ms depolarizing steps to various potentials ranging from –80 to +40 mV in 5-mV increments. B, N395K did not alter steady-state inactivation. Steady-state inactivation was estimated using a standard double pulse protocol in which sodium currents were induced by a 50-ms depolarizing potential of 0 mV following a 500-ms prepulse at potentials that ranged from -150 to +10 mV with a 10-mV increment. Cells were held at –120 mV. All curves were fitted to a Boltzmann function. $V_{1/2(activation)}$: rpNav1.7, -26.6 ± 0.9, *n* = 5 *vs* N395K, -36.9 ± 1.0, *n* = 4, p = 0.0918. $V_{1/2(inactivation)}$: rpNav1.7, -79.1 ± 1.3, *n* = 5 *vs* N395K, -81.4 ± 0.9, *n* = 4, p = 0.6119.
